# Patient‐Physician Agreement on Diagnosis in the ED, Part of Diagnostic Excellence. A Prospective Multicenter Study

**DOI:** 10.1111/acem.70365

**Published:** 2026-06-17

**Authors:** Lieke Claassen, Veerle M. E. P. Baars, Jochen W. L. Cals, Patricia M. Stassen, Gideon H. P. Latten

**Affiliations:** ^1^ Department of Emergency Medicine Zuyderland Medical Centre Heerlen the Netherlands; ^2^ Department of Family Medicine, Care and Public Health Research Institute (CAPHRI) Maastricht University Maastricht the Netherlands; ^3^ Division General Medicine, Department of Internal Medicine, Section Acute Medicine Maastricht University Medical Center Maastricht the Netherlands; ^4^ Cardiovascular Research Institute Maastricht (CARIM) Maastricht University Maastricht the Netherlands; ^5^ Department of Emergency Medicine Maastricht University Maastricht the Netherlands

An important aspect of medicine is making a timely and accurate diagnosis. Sometimes, however, errors occur. Where diagnostic error was previously mainly seen as misdiagnosis by the physician, the National Academies of Sciences, Engineering and Medicine redefined diagnostic error as “a failure to establish an accurate and timely explanation of the patient's health problem(s) or to communicate that explanation to the patient” [1, 2]. One could argue that diagnostic excellence is therefore not only making the right diagnosis, but also establishing agreement on the diagnosis between patient and physician.

In the emergency department (ED), achieving diagnostic excellence is challenging. Physicians primarily aim to exclude life‐threatening conditions rather than establish an definitive diagnosis [3] and they do so under time pressure within a single visit without an established patient‐physician relationship. These factors likely complicate the diagnostic process, and also influence the patient's understanding of the diagnosis [3].

Previous research on agreement of patient‐physician diagnosis has mainly focused on (specific) diseases in non‐urgent settings. In these studies, agreement varied widely (32%–74%) [4, 5]. However, agreement in the ED in patients with no prespecified diagnoses (i.e., unselected patients) remains scarcely studied [6]. While striving for diagnostic excellence, it is important to investigate the diagnosis agreement in the ED as well.

In this prospective study, we examined diagnostic agreement of unselected adult ED patients in the Netherlands and factors associated with agreement. This is a secondary analysis of a 72‐h prospective study investigating the journey of unselected adult (≥ 18 years) ED patients across six EDs in a Dutch province (1.1 million inhabitants), details in [7]. Files with missing data on the diagnosis (by either patient or physician) were excluded.

Patient data were collected by trained researchers through a questionnaire completed by the patients and/or with help of their caregivers (patient diagnosis) and a questionnaire completed by the physician and/or utilizing information from medical records (physician diagnosis). The question in the patient's questionnaire:

“What diagnosis did the doctor made at discharge from the ED?” was answered by the patient or his/her caregivers during their ED stay. The question in the physicians questionnaire: “What was the diagnosis at discharge from the ED?” was answered after the ED visit or later by the researcher based on the medical records. It was expected that due to crowding, it would not always be feasible to address this question at the end of the ED stay when the final diagnosis was communicated, resulting in missing data.

Agreement was defined as the level of concordance between the diagnoses independently reported by patients and physicians at ED discharge. Responses were classified as being in agreement when they reflected the same or a closely related diagnosis (e.g., “COVID pneumonia” vs. “bilateral pneumonia,” “wrist fracture” vs. “scaphoid fracture,” or “rhythm disorder” vs. “atrial fibrillation”). All responses were independently evaluated by two experienced clinicians (GL and LC). In case of disagreement, consensus was reached after discussion. Agreement was assessed percentage total agreement. Pearson's chi‐square or Fishers Exact tests were used to compare categorical variables between the agreement and disagreement group with Cramer's V to quantify the strength of the association. Mann Whitney *U* test was used to compare continuous variables. Odds ratios were calculated for variables associated with agreement. A sensitivity analysis was performed to investigate the impact of missing data, and some characteristics were compared (sex, age, level of education, living situation, triage urgency, work‐up, surgical vs. non‐surgical condition, ED‐LOS, hospital admission and adverse outcome). Statistical significance was set at *p* < 0.05. All statistical analyses were performed using IBM SPSS statistical software version 28 (Chicago, Illinois, USA). The study was reviewed and approved by the medical ethics committee of Zuyderland (METC‐Z nr. 20,210,142) and boards of directors of all participating hospitals.

Of the 583 included patients, 351 (60%) had complete data on both study questions and were included in the final analysis. Detailed reasons for missing data on diagnoses were not systematically recorded due to the multicenter design. Based on study team experience, missing data were most likely due to interviewing patients relatively early in the ED process. Sensitivity analysis found significantly longer ED‐LOS and more complex work‐up in excluded patients (Table 1), although absolute differences were small.

**TABLE 1:** Comparison of patients with and without diagnosis agreement (*n* = 351).

	Diagnosis agreement (*n* = 303, 86.3%)	Diagnosis disagreement (*n* = 48, 13.7%)	*p*
	*n* (%)	*n* (%)	
Sex: Female	146 (48.2%)	24 (50.0%)	0.815
Age (med, years)	65 (IQR 46–77)	70 (IQR 39–78)	0.777
Highest level of education (*n* = 350)			0.256
No education	6 (2.0%)	1 (2.1%)	
Primary school	24 (7.9%)	5 (10.4%)	
Secondary school	116 (38.3%)	12 (25.0%)	
Tertiary education	156 (51.5%)	30 (62.5%)	
Living situation			0.270
Independent living	252 (83.2%)	38 (79.2%)	
Domiciliary care/assisted living	36 (11.9%)	5 (10.4%)	
Other	15 (4.9%)	5 (10.4%)	
Triage urgency (*n* = 346)^a^			0.218
Low	256 (84.5%)	36 (78.3%)	
High	44 (14.5%)	10 (21.7%)	
Work‐up^b^			< 0.001*
Simple	191 (63.0%)	18 (37.5%)	
Complex	112 (37.0%)	30 (62.5%)	
Condition			< 0.001*
Surgical	161 (53.1%)	12 (25.0%)	
Non‐surgical	142 (46.9%)	36 (75.0%)	
ED‐LOS (med, minutes) (*n* = 350)	161 (IQR 103–241)	212 (IQR 166–278)	< 0.001*
Short ≤ 3 h	174 (57.4%)	16 (33.3%)	0.003*
Hospital admission	138 (45.5%)	27 (56.3%)	0.167
Adverse outcome^c^	50 (16.5%)	8 (16.7%)	0.977

*Note:* *p < 0.05.

Abbreviation: ED‐LOS, emergency department length of stay.

^a^ *Triage:* triage urgency levels were determined according to the MTS. Red and orange were combined as high, and yellow, green and blue as low.

^b^ *Work‐up:* Simple: laboratory testing, ECG, conventional radiography. Complex: CT, ultrasound, MRI, lumbar puncture, abdominal paracentesis, endoscopy and consultation of other specialties.

^c^ *Adverse outcome:* Deceased or ED revisit within 30 days.

The median age in the study population was 65 years (IQR 45–77) and 48.4% of patients were female. The absolute agreement on the diagnosis was 86.3%, indicating a high level of observed concordance [8].

Compared to the disagreement group, the agreement group more often had surgical conditions (53.1% vs. 25.0%, *p* < 0.001, Cramer's *V* = −0.193), a simple work‐up (laboratory testing, ECG and conventional radiography) (63.0% vs. 37.5%, *p* < 0.001, Cramer's *V* = 0.179) and a short ED‐LOS (≤ 3 h) (57.4% vs. 33.3%, *p* = 0.003, Cramer's V = 0.168). No significant associations were found between agreement and sex, age, highest level of education, living situation, triage urgency, hospital admission and adverse outcome.

The odds ratio (OR) for agreement in patients with a surgical condition was 3.40 (95% CI: 1.70–6.79), for a simple work‐up 2.84 (95% CI: 1.52–5.33), and for a short ED‐LOS (≤ 3 h) the OR was 2.72 (95% CI 1.43–5.17).

In summary, the absolute agreement on diagnosis between patients and physicians in unselected adult ED patients was 86.3%. This is one of the first studies investigating this agreement between patients and physicians in the ED. We observed that factors associated with agreement were presentation with a surgical condition, a simple diagnostic work‐up, and a short ED‐LOS. These 3 factors were associated with a threefold higher odds of agreement in diagnosis. No significant associations were found between agreement and the other variables.

The high agreement (86.3%) we found is noteworthy, particularly when compared to studies in non‐ED settings that reported agreement varying from 32% to 74% [4, 5]. The fact that our observed agreement was higher is interesting, taking the challenges in the diagnostic process in the ED into account [2]. It may be possible that our agreement is somewhat overestimated because missing patients more often had a longer ED‐LOS and complex work‐up than included patients, but differences were only slight.

High agreement in our study population may be explained by rapid, focused diagnostic testing, resulting in quicker answers than the longer outpatient diagnostic process. A short‐time span may lead to better understanding of the diagnosis as no information is lost over time. High agreement may also reflect that agreement is greater when communicating major health problems and new diagnoses, which often applies to the ED [4].

A higher agreement was found in patients with surgical conditions, possibly because the ED diagnosis established in these patients such as fractures or wounds is easier to understand than complex neurological or medical diagnoses. Besides, there was an association between a simple work‐up and shorter ED‐LOS. Surgical complaints generally involve shorter ED‐stays and less complex work‐up as well [9]. A lower complexity, in general, may result in less complex matters to explain, and therefore, to higher agreement.

Despite being one of the few to investigate this topic and the strength associated with its multicenter design, our study has some limitations.

Analysis of agreement was not the primary research question and, although included in the questionnaire, the focus during data collection was on the characteristics and patient journey of the patients. We could not retrieve a diagnosis from both physician and patient in all cases. As differences in wording between patients and physicians may reflect nuances in communication rather than true lack of understanding, we based agreement on interpreted meaning rather than exact wording. While our measure captures concordance in reported diagnoses, this loss of nuance should be taken into account when interpreting the findings. Finally, it is important to note that we did not take other factors that may influence agreement into account, such as physician‐level characteristics (e.g., experience or sex) or the presence of caregivers that could influence agreement.

Diagnostic excellence is not only establishing the correct diagnosis, but also communicating it clearly. Our data can help improve the patient‐physician agreement on the diagnosis. Getting insights into these factors associated with agreement will contribute to diagnostic excellence, to less diagnostic errors, and an increase in patient satisfaction. Further research is, however, needed.

In conclusion, in unselected adult ED patients, we found high agreement in diagnosis between patient and physician, especially in those with surgical conditions, simple diagnostic work‐up, and short ED‐LOS. This study showed that, despite the setting of working under time pressure in acute situations and without an established patient‐physician relationship, it is still possible to establish an accurate explanation of the diagnosis and to communicate that clearly to the patient. Communication is an important aspect in achieving diagnostic excellence, and all physicians need to be aware of the groups (those with medical conditions, complex diagnostic work‐up, and long ED‐LOS) that are prone to not getting an accurate and timely explanation of their health problem.

## Author Contributions

L.C., G.H.P.L., and P.M.S. contributed to the study concept and design, interpretation of the data, drafting of the manuscript, and critical revision. V.M.E.P.B. and L.C. contributed to data acquisition and interpretation of data. J.W.L.C. contributed to the interpretation of the data and critical revisions. All authors approved the final version of the manuscript.

## Funding

The authors are grateful to acknowledge the support of the SGO fund (Dutch Emergency Medicine Fund), which provided funding for this study.

## Conflicts of Interest

The authors declare no conflicts of interest.

## Data Availability

The data that support the findings of this study are available on request from the corresponding author. The data are not publicly available due to privacy or ethical restrictions.

## References

[1] T. D. Giardina , H. Hunte , M. A. Hill , S. L. Heimlich , H. Singh , and K. M. Smith , “Defining Diagnostic Error: A Scoping Review to Assess the Impact of the National Academies' Report Improving Diagnosis in Health Care,” Journal of Patient Safety 18, no. 8 (2022): 770–778. 35405723 10.1097/PTS.0000000000000999 PMC9698189

[2] P. Mahajan , “From Diagnostic Errors to Diagnostic Excellence in Emergency Care: Time to Flip the Script,” Academic Emergency Medicine 32, no. 3 (2024): 366–368. 39428624 10.1111/acem.15033 PMC11921061

[3] A. M. Doty , K. L. Rising , T. Hsiao , et al., “Unfortunately, I Don't Have an Answer for You: How Resident Physicians Communicate Diagnostic Uncertainty to Patients During Emergency Department Discharge,” Patient Education and Counseling 105, no. 7 (2022): 2053–2057. 35168855 10.1016/j.pec.2021.12.002 PMC9177889

[4] S. M. Scheitel , B. J. Boland , P. C. Wollan , and M. D. Silverstein , “Patient‐Physician Agreement About Medical Diagnoses and Cardiovascular Risk Factors in the Ambulatory General Medical Examination,” Mayo Clinic Proceedings 71, no. 12 (1996): 1131–1137. 8945482 10.4065/71.12.1131

[5] N. Verma , B. Buch , R. Taralekar , and S. Acharya , “Diagnostic Concordance of Telemedicine as Compared With Face‐To‐Face Care in Primary Health Care Clinics in Rural India: Randomized Crossover Trial,” JMIR Form Res 23, no. 7 (2023): e42775. 10.2196/42775 PMC10337309 37130015

[6] A. N. Makaryus and E. A. Friedman , “Patients' Understanding of Their Treatment Plans and Diagnosis at Discharge,” Mayo Clinic Proceedings 80, no. 8 (2005): 991–994. 16092576 10.4065/80.8.991

[7] L. Claassen , P. M. Stassen , T. J. T. Boumans , et al., “Characteristics of Dutch ED Patients and Their Journey Through the Acute Care Chain: A Province‐Wide Flash‐Mob Study,” PLoS One 20, no. 4 (2025): e0318510. 40179077 10.1371/journal.pone.0318510 PMC11967924

[8] J. R. Landis and G. G. Koch , “The Measurement of Observer Agreement for Categorical Data,” Biometrics 33, no. 1 (1977): 159–174. 843571

[9] D. van der Veen , C. Remeijer , A. J. Fogteloo , C. Heringhaus , and B. de Groot , “Independent Determinants of Prolonged Emergency Department Length of Stay in a Tertiary Care Centre: A Prospective Cohort Study,” Scandinavian Journal of Trauma, Resuscitation and Emergency Medicine 26, no. 1 (2018): 81. 30236125 10.1186/s13049-018-0547-5 PMC6148782

